# Fabrication and Encapsulation of Soy Peptide Nanoparticles Using Ultrasound Followed by Spray Drying

**DOI:** 10.3390/foods13233967

**Published:** 2024-12-09

**Authors:** Yiqun Jiang, Zhen Luo, Fenglan Xiang, Yubin Liu, Jin Yan, Jinmei Wang

**Affiliations:** 1 National Engineering Laboratory of Wheat & Corn Further Processing, School of Food Science and Engineering, South China University of Technology, Guangzhou 510640, China; jiangyiqunaz@163.com (Y.J.);; 2 Infinitus (China) Co., Ltd., Guangzhou 510623, China

**Keywords:** insoluble peptide aggregates, ultrasound, spray drying, bitter taste, encapsulation

## Abstract

Peptide aggregation inevitably occurs during hydrolysis, and insoluble peptide aggregates (ISPA) are used as feed for animals due to their poor water solubility and unpleasant bitter flavor. Ultrasound was used to fabricate soy peptide nanoparticles by reassembling ISPA, followed by spray-drying encapsulation to develop low-bitterness peptide microcapsules with soluble soybean polysaccharide (SSPS) and stevioside (STE) as wall materials. Powder properties, bitter taste, and the morphology of the microcapsules were evaluated. The formation of soluble peptide nanoparticles (<200 nm) was observed after ultrasound due to the reassembly of ISPA through the disruption of non-covalent intermolecular interactions. A gradual reduction in bitter taste was observed with increasing ultrasonic time. Moreover, spray-drying encapsulation with STE could effectively improve the flowability and wettability of the microcapsule powder owing to the rapid migration of surface-active STE to the atomized droplet surface, as evidenced by the lower angle of repose and wettability time. Peptide microcapsules with STE (spherical particles with smooth surfaces) exhibited lower density and reduced bitterness because STE (0–0.1%, *w*/*w*) exhibited an excellent bitter-masking effect. With high STE concentrations (>0.5%, *w*/*w*), microcapsules exhibited a higher bitter taste than unencapsulated peptides due to the increased surface distribution of STE on the microcapsules. These results provide an effective technique to improve the physicochemical properties of ISPA.

## 1. Introduction

To prepare bioactive peptides, the formation of insoluble peptide aggregates inevitably occurs during the protease hydrolysis of soy protein. Moreover, hydrophobic interactions and disulfide bonds between peptide-peptide/protein are further enhanced following subsequent heat inactivation. The insoluble aggregates are usually thrown away or used as feed for animals. However, these insoluble aggregates with a high proportion of essential amino acids have good digestibility [1]. Moreover, the digestive products exhibited excellent antioxidant activity owing to the appearance of bioactive peptides [1].

Due to the poor solubility and unpleasant bitter taste of insoluble peptide aggregates, there has been little research focused on these topics. Recently, ultrasonic treatment or pH-shifting treatment have been used to prepare peptide-based nanoparticles from insoluble soy peptide aggregates. The cavitation effect of ultrasound can generate powerful shear and mechanical forces, leading to the disruption of internal molecular interactions [2]. These nanoparticles have been used as carriers for delivering hydrophobic active ingredients. The water dispersibility and bioaccessibility of curcumin loaded into peptide-based nanoparticles were remarkably enhanced [3,4]. Meanwhile, peptide nanoparticles have good emulsifying and foaming abilities and can act as stabilizers in emulsions or creams [5]. However, insoluble peptide aggregates may be always perceived with a significant bitter taste because of the appearance of hydrophobic amino acids. It seems to be attributed to the exposure of hydrophobic amino acids of peptides, especially Leu, Phe, and Tyr residues [6].

Debittering processes, such as isoelectric point precipitation, chromatographic separation, activated carbon adsorption, selective extraction with organic solvents, enzymatic and microbial debittering, and masking methods, have been attempted to improve the bitter taste of peptides [6,7]. Furthermore, encapsulation technologies, including coacervation, spray drying, extrusion, supercritical fluid extraction, cocrystallization, and inclusion, have also been widely applied to mask bitterness and improve peptide stability [8]. Spray drying is the most extensively used one in the food industry because of its lower cost compared to freeze-drying or coacervation, and spray-dried microcapsules usually have good product stability. Spray drying has been used to encapsulate bitter peptides and protect functional components using polysaccharides, proteins, fats, and other compounds as wall materials [9]. Spray drying with gum Arabic, maltodextrin, and soy protein isolate has been attempted to reduce the hygroscopicity and bitter taste of protein hydrolysates [10,11,12,13]. Polysaccharides are widely used as encapsulating wall materials owing to their advantages of stable structure, abundant natural resources, and low cost. However, using only polysaccharides as wall materials can lead to more concavities on microspheres, reduced powder flowability, and lower encapsulation efficiency of peptide microcapsules due to rapid dehydration during the spray-drying process [14]. The low molecular weight surfactants (e.g., Tween-80) were introduced to modify the morphology of the microcapsule by their competitive surface migration with peptides [15,16].

In this study, ultrasound followed by spray drying was used to develop low-bitterness peptide microcapsules from insoluble peptide aggregates. The ultrasound-induced reassembly of peptide nanoparticles was investigated. Soluble soybean polysaccharide (SSPS), a low-viscosity, negatively charged polysaccharide, could stabilize soy protein and fabricate stable nanogels and emulsions across a wide pH range [17,18,19]. In this study, SSPS was chosen as the wall material to encapsulate peptide nanoparticles using spray drying. In addition, sweetener stevioside (STE) was also introduced to modify the bitterness and morphology of the microcapsules because STE exhibits interfacial activity due to its amphiphilic structure, consisting of a hydrophobic steviol backbone and hydrophilic sugar residues [20]. Thus, the physicochemical properties of spray-dried ISPA microcapsules, including product yield, encapsulation efficiency, bulk density, angle of repose, wettability, bitterness, and morphological structure, of microcapsule powders were examined.

## 2. Materials and Methods

### 2.1. Materials

The defatted soybean flour was obtained from Yuxin Biotechnology Co., Ltd. (Binzhou, China). Alcalase (alkaline protease) was supplied by Novozymes (Copenhagen, Denmark). STE was purchased from Jining Aoxing Stevia Products Co., Ltd. (Jining, China). SSPS was obtained from Weibo Foods Co., Ltd. (Quanzhou, China). The Folin phenol reagent kit was purchased from Beijing Dingguo Company (Beijing, China). The ANS (8-Anilino-1-naphthalenesulfonic acid) was purchased from Shanghai Macklin Biochemical Technology Co., Ltd. (Shanghai, China). Quinine was purchased from Aladdin (Shanghai, China). All other chemicals were of analytical grade.

### 2.2. Protein Hydrolysis

Soy protein isolate (SPI) was prepared using alkaline extraction (pH 8.0) and precipitation at pH 4.5, as described by Wang et al. [21]. SPI was dispersed in distilled water (4%, *w*/*v*) and subjected to Alcalase hydrolysis (1% *w*/*w*, protein basis, pH 8.0, 60 °C). After 4 h of hydrolysis, the protein hydrolysates were immediately adjusted to pH 7.0 using 1.0 mol/L HCl and heat-inactivated (boiling water bath, 10 min). The protein hydrolysate was then centrifuged at 8000× *g* for 20 min and the obtained precipitate (ISPA) was lyophilized (CHRIST-Alpha 1–4 LD plus, Osterode, Germany).

### 2.3. Intermolecular Interaction Analysis

The ISPA was dissolved in the following six solvents: (1) Phosphate buffer solution (PBS): 10 mM phosphate buffer, pH 7.0; (2) Dithiothreitol (DTT) solution: PBS solution containing 5 mM DTT; (3) urea: PBS solution containing 6 M urea; (4) Sodium dodecyl sulfonate (SDS) solution: PBS solution containing 1% (*w*/*v*) SDS; (5) SDS-urea: PBS solution containing 6 mol/L urea and 1% (*w*/*v*) SDS; (6) SDS-urea-DTT: PBS solution containing 6 mol/L urea, 1% (*w*/*v*) SDS, and 0.05 mol/L DTT. The insoluble aggregates were dispersed in the above solvents (2 mg/mL), stirred for 5 h at room temperature, and then centrifuged (8000× *g*, 20 min). The protein content in the supernatant was determined by the Lowry method. The protein content of the sample powder was determined by the Kjeldahl method. The protein solubility was expressed as protein content in supernatant/protein content in sample powder.

### 2.4. Encapsulation of Peptide Nanoparticle Using Ultrasound Followed by Spray Drying

Insoluble aggregates were dispersed in distilled water (1%, *w*/*v*) and adjusted to pH 7.0. The dispersions were sonicated for 0–30 min using a SNIC RUPTOR250 ultrasonic processor (Omni International, Kennesaw, GA, USA) with a 10 mm probe (150 W, 20 kHz). An ice bath was used to keep the temperature of the dispersion at 25 °C. The size distribution of nanoparticle dispersions was monitored using dynamic light scattering (DLS) with a Zetasizer Nano ZS instrument (Malvern Instruments, Malvern, UK). Samples were placed in a 1 cm × 1 cm cuvette (PCS8501) and analyzed at a fixed scattering angle of 173° at 25 °C. The refractive index of water was 1.33 and three independent DLSs were performed for each sample run.

STE and SSPS were added to the peptide nanoparticle solution (sonicated for 20 min) to achieve final concentrations of 0–1.0% (*w*/*w*) and 10% (*w*/*w*), respectively. Wall and core materials (peptide nanoparticles) were mixed at a ratio of 1:1 (*v*/*v*). The pH of the solution was adjusted to 7.0 before spray drying. A spray dryer (B-290, Büchi, Flawil, Switzerland) was used, with a peristaltic pump flow rate of 4 mL/min. The inlet and outlet air temperatures were set to 170 °C and 90 °C, respectively. Spray-dried microcapsule powders were stored at room temperature.

### 2.5. Fluorescence Spectroscopy

The ANS solution (10 μL, 5.0 mM, pH 7.0) was added to 1 mL of the sample solution (0.5 mg/mL) and mixed well. The excitation wavelength was set to 390 nm, and the emission wavelength range was 420–600 nm. The emission wavelength was scanned using a fluorescence spectrophotometer (Hitachi Co., Tokyo, Japan).

### 2.6. Bitter Taste Evaluation

Ten trained panelists (five males and five females, aged between 20 and 35 years) evaluated the bitter taste of the samples. Standard solutions of quinine were used at concentrations of 0, 4, 8, 12, 16, and 20 μg/mL, which were scored as 0, 2, 4, 6, 8, and 10, respectively. A 2% (*w*/*v*) microcapsule dispersion was prepared, one at a time in a tasting cup, coded with a three-digit random number. Panel members were asked to rinse their mouths between samples. Sensory evaluations were conducted in individual compartments on a laboratory scale under red light at room temperature (25 °C).

### 2.7. Powder Physical Analysis

The microcapsule dispersion (2%, *w*/*v*) was centrifuged (8000 rpm, 10 min). Encapsulation efficiency (EE) was evaluated by determining the peptide content of supernatants by Lowry’s method, and could be was expressed as free peptides content in supernatant/total peptide content. The product yield was calculated based on the ratio of recovered powder to the total sample content.

Scanning electron microscopy (SEM, JEM-1400F, JEOL, Tokyo, Japan) was used to assess the morphological structure of the samples. The sample was photographed under vacuum after coating with 10 nm gold-palladium. The SEM was operated at a voltage of 5 kV.

Powder (2 g) was poured into a 10 mL measuring cylinder to determine both bulk density (BD) and tapped density (TD). The cylinder was struck every two seconds for four times at a height of 2 cm from the table. The volume of powder at this point was recorded as the bulk volume. Tapping was continued until the volume remained constant, and this was recorded as the tapped volume. Densities were calculated by dividing the powder weight by the volume. The compressibility index (CI) of powder was calculated using the following formulas:

“CI (%) =“ [“TD − BD”/“TD”]”×100”(1)

Wettability was expressed as the average time required for 0.1 g of powder to be completely immersed in 8 ml of water. Eight milliliters of room-temperature water were added to a 10 mL beaker (25 mm diameter), and 0.1 g of microcapsule powder was poured in all at once. The sample was observed for infiltration and the time to complete infiltration was recorded. A cone was obtained by pouring 10 g of powder from a height of 5 cm into a funnel with an outlet diameter of 12 mm. The angle formed between the side and the bottom of this vertebra represented the angle of repose [22].

### 2.8. Statistical Analysis

All experiments were performed in triplicate. An analysis of variance (ANOVA) was conducted on the data within groups, and significant differences were determined using Duncan’s test at *p* < 0.05. Statistical analysis was carried out using SPSS 20.0 (SPSS Inc., Chicago, IL, USA).

## 3. Results and Discussion

### 3.1. Enzymatic-Induced Aggregation of Soybean Protein

**Figure 1:**
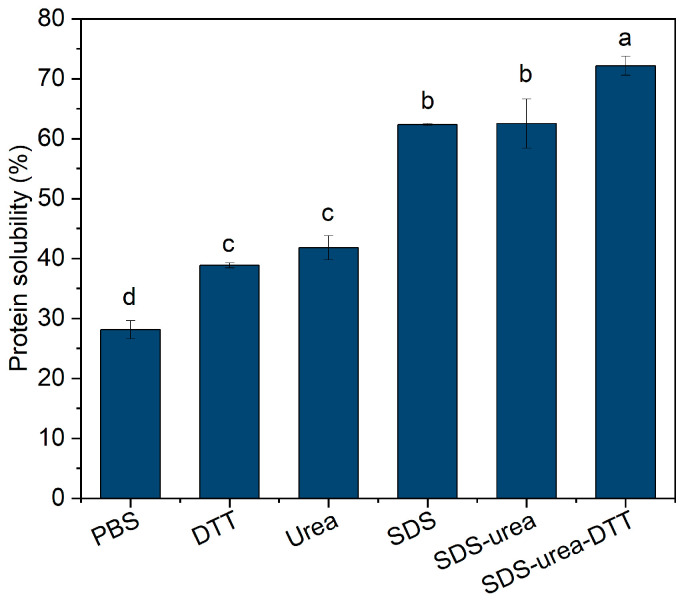
Intermolecular interaction involved in insoluble soy peptide aggregates. Different letters represent significant differences (*p* < 0.05) between different samples.

After 4 h of enzymatic treatment, 14% of insoluble peptide aggregates (ISPA) were formed. To clarify the types of intermolecular forces in ISPA, a reductive dissolution method was employed. Five different reducing agents or combinations thereof were used to selectively disrupt specific intermolecular forces within ISPA. Hydrophobic interactions, disulfide bonds, and hydrogen bonds were mainly disrupted by SDS, DTT, and urea, respectively [23,24]. This experiment determined the contribution of different interactions to peptide aggregation by evaluating the solubility of ISPA in dissolution solvents. As shown in Figure 1, adding DTT, urea, SDS, or their combinations to PBS buffer obviously increased water solubility of ISPA, indicating that both covalent (disulfide bonds) and non-covalent (electrostatic interactions, hydrophobic interactions, and hydrogen bonds) interactions stabilized the insoluble aggregates. ISPA showed the highest protein solubility in SDS-PBS, followed by urea-PBS and DTT-PBS, indicating a predominant hydrophobic interaction stabilized the structure of ISPA. Hydrogen bonds and disulfide bonds were also involved in stabilizing ISPA. Similar findings were reported by Zhang et al. [1].

### 3.2. Ultrasound-Induced Soluble Peptide Nanoparticles

**Figure 2:**
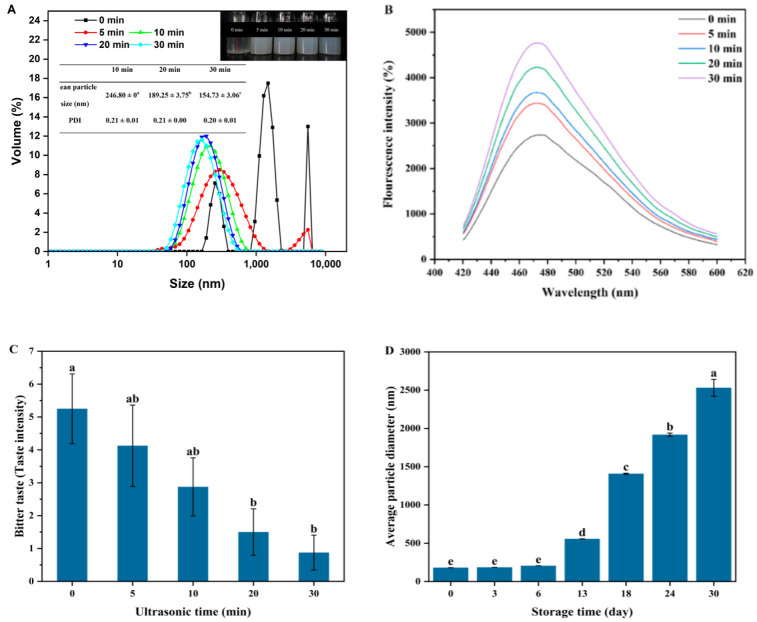
(**A**) Influence of ultrasound time on the particle size distribution of ISPA (Inset: Visual observations and the mean particle size of peptide nanoparticle dispersions); (**B**) Fluorescence spectra and bitter taste intensity (**C**) of peptide nanoparticle dispersions after ultrasonic treatment; (**D**) Average particle diameter of peptide nanoparticles treated with ultrasound for 20 min during storage at 25 °C. Different letters represent significant differences (*p* < 0.05) between different samples.

High-intensity ultrasound was used to induce the unfolding and reassembly of ISPA, as shown in Figure 2. ISPA (0 min) tended to precipitate when it was dispersed in distilled water. A large particle with a wide size distribution was observed for ISPA (Figure 2A, inset). After ultrasonic treatment, a uniform peptide dispersion was observed (Figure 2A). The turbidity and particle size of insoluble aggregate dispersions significantly decreased with increasing ultrasonic time, which might be attributed to the fact that high-intensity ultrasound disrupted non-covalent interactions (e.g., hydrophobic interactions and hydrogen bonds), converting large aggregates into smaller particles. The size of peptide nanoparticles was less than 200 nm after long ultrasound treatment (>20 min). To further investigate the ultrasound-induced conformation of insoluble peptide nanoparticles, we examined the extrinsic fluorescence spectra of peptide nanoparticle dispersions (Figure 2B). An increase in ANS fluorescence intensity was observed with increasing ultrasonic time, suggesting increased surface hydrophobicity. Ultrasonic treatment could induce molecular unfolding of ISPA by destroying hydrophobic interactions of insoluble peptide aggregates (Figure 1). Some previous studies have shown that the surface hydrophobicity of proteins significantly increases after ultrasonic treatment [25,26]. More hydrophobic groups (e.g., Tyrosine, Tryptophan, and Phenylalanine) and regions inside peptide aggregate molecules were exposed outside, increasing surface hydrophobicity of soluble peptide nanoparticles. It seems that an increase in surface hydrophobicity was not consistent with the increased ultrasound-induced solubilization of ISPA. In fact, ultrasound-induced reassembly of insoluble peptide nanoparticles leads to synchronous exposure of hydrophobic and hydrophilic groups to the polar surrounding environment. The balance of hydrophobic and hydrophilic sites was possibly thus responsible for the increased solubility of peptide nanoparticles.

Remarkably, the bitter taste intensity of ISPA dispersions continuously and significantly decreased with increasing ultrasonic time (Figure 2C), consistent with the reduction in peptide aggregate size. This phenomenon may be attributed to ultrasound-induced reassembly of insoluble aggregates and the formation of soluble peptide nanoparticles (Figure 2A). The bitter taste of peptides is generally considered to be associated with the average hydrophobicity, amino acid sequence, molecular weight, and steric conformation [7]. It seems to be inconsistent with the fact that ultrasound-induced peptide nanoparticles have higher surface hydrophobicity (Figure 2B). In fact, during ultrasound-induced reassembly, both hydrophobic and hydrophilic groups are exposed. Bitter groups may be embedded in the core of peptide nanoparticles, and/or the binding of hydrophobic bitter amino acids to bitter receptors may be partially shielded by hydrophilic groups. Therefore, decreased presence of bitter groups on the particle surface may be a reasonable explanation for the reduction in bitter taste although the surface hydrophobicity of proteins significantly increases (Figure 2B).

The ultrasound-induced peptide nanoparticle dispersion was stored at 25 °C for a month to evaluate storage stability, and the particle size of peptide nanoparticles was monitored (Figure 2D). An obvious increase in particle size after 6 days was observed (Figure 2D), suggesting unstable re-aggregation of soluble peptide nanoparticle during the storage process. The high surface hydrophobicity of peptide nanoparticles may be the main reason for this instability (Figure 2B). Intermolecular hydrophobic interactions of peptide nanoparticles resulted in the formation of larger particles [3,4,5]. The instability of peptide nanoparticles may not be helpful for the application of industrial liquid food products although bitter taste intensity of soluble peptide nanoparticle significantly reduced. Thus, the improved physical stability of peptide nanoparticles is desired, and the encapsulation of peptide nanoparticles may be an effective strategy.

### 3.3. Encapsulation of Peptide Nanoparticles

**Figure 3:**
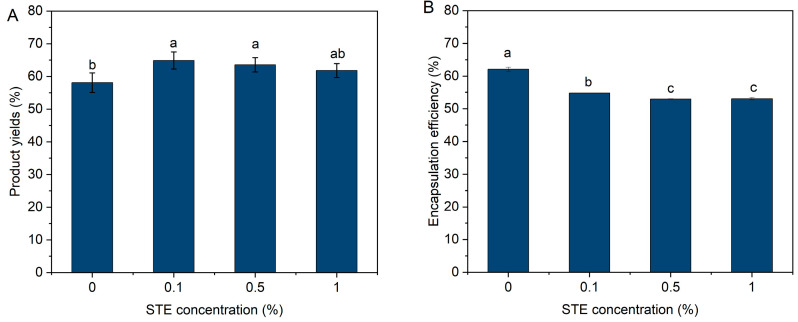
Product yields (**A**) and encapsulation efficiency (**B**) of peptide microcapsules with different STE concentrations. Different letters represent significant differences (*p* < 0.05) between different samples.

**Figure 4:**
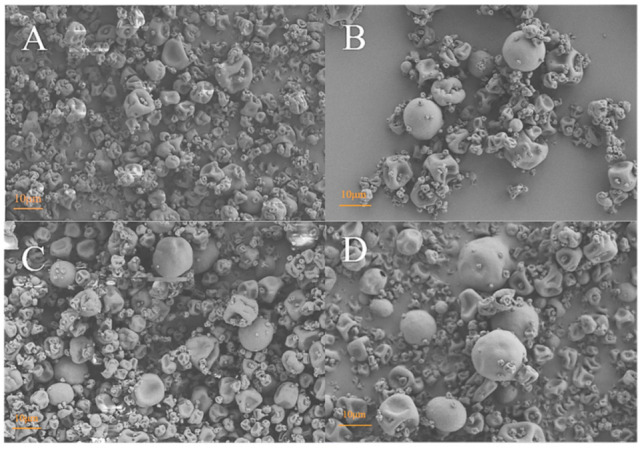
SEM images of (**A**–**D**) peptide microcapsules with different STE concentrations (0, 0.1%, 0.5%, 1%, *w*/*w*).

To further reduce the bitter taste and improve the physical stability of ultrasound-induced nanoparticles, spray drying was used to encapsulate peptide nanoparticles using SSPS and STE as wall materials. SSPS as the wall material may provide more steric hindrance around peptide nanoparticles to resist their re-aggregation. Wan et al. verified that STE exhibited interfacial activity due to its amphiphilic structure (hydrophobic steviol backbone and hydrophilic sugar residues) [20]. The addition of amphiphilic STE may benefit peptide nanoparticle microencapsulation, aside from its sweetness masking bitterness. The product yield of peptide microcapsules increased with increasing STE concentrations (Figure 3A). The increase in product yield for peptide microcapsules may be related to improved powder mobility during spray drying, as evidenced by the decreased angle of repose (Table 1). Microcapsule powder particles with smoother surfaces and larger sizes showed lower particle adhesion to each other and the wall chamber (Figure 4), making it easier to blow out the powder and thus increasing product yields (Figure 3A). Wan et al. have verified that STE shows high interfacial activity due to its amphiphilic structure. Therefore, during the atomization and spray-drying process, surfactant molecules (STE) can preferentially migrate to the air/water interface and mainly distribute on the surface of peptide microcapsules [27]. However, the addition of STE led to a significant decrease in encapsulation efficiency (Figure 3B). The preferential migration of STE probably hindered the migration of another macromolecular wall material (SSPS) to the microcapsule surface, resulting in a significant decrease in peptide encapsulation efficiency (Figure 3B).

Spray-dried peptide microcapsules with only SSPS as the wall material exhibited wrinkled particles with dents on their surface (Figure 4A). Adding STE to the wall material increased the particle size of peptide microcapsules. changes in the shapes and surface structures of particles were also observed (Figure 4B–D). With increasing STE concentrations, more spherical particles with smooth surfaces and larger sizes were observed. This phenomenon could be related to the preferential migration of surface-active STE molecules onto the surface of droplets during atomization and drying, which credibly explained the increase in microcapsule product yields (Figure 3A). The use of STE through competitive surface migration with peptides led to the formation of spherical particles with relatively smooth surfaces. Sarabandi and Jafari also found that the introduction of Tween 80 resulted in morphological changes in spray-dried flaxseed peptide microcapsules [15]. The transformation of wrinkled structures to smooth-surfaced spherical particles was similarly observed for protein nanoparticles (bovine serum albumin as a protein model) [28].

The powder properties (wettability, angle of repose, bulk density, and tap density) of microcapsule powders with different STE concentrations were analyzed, as shown in Table 1. Compared to microcapsule powders without STE, smaller bulk density and tap density of particles were observed for peptide microcapsules with different STE concentrations, indicating higher porosity. These results are in agreement with the morphological structure of powder particles (Figure 4).

The addition of STE resulted in significant decreases in the angle of repose (from 48.47° to 40.29°) and wettability (from 586.00 s to 300.67 s) (Table 1), suggesting a reduction in the contact area and friction force between adjacent microcapsule particles [16,29]. A reasonable explanation is the preferential migration of surfactant molecules to the droplet/particle surface during the atomization and drying process [15]. Meanwhile, flexible SSPS and STE quickly migrated to the surface of the droplet, accelerating the formation of a glassy film around the droplet [27,30]. This film may be capable of overcoming the coalescence of droplets and reduce wall stickiness. Thus, microcapsule powder can be blown out more easily, followed by improving production yield (Figure 3). Peptide microcapsules with higher porosity penetrated water more quickly, which is responsible for the increase in wettability with increasing STE concentrations (Table 1). In addition, wettability is influenced by many other factors such as hygroscopicity and particle size [31]. Although no significant differences in hygroscopicity were observed for microcapsule powders, the morphological changes of microcapsule powders may provide another explanation for increased wettability. Larger particle sizes and reduced agglomeration were observed for microcapsule powders after adding STE (Figure 4).

**Figure 5:**
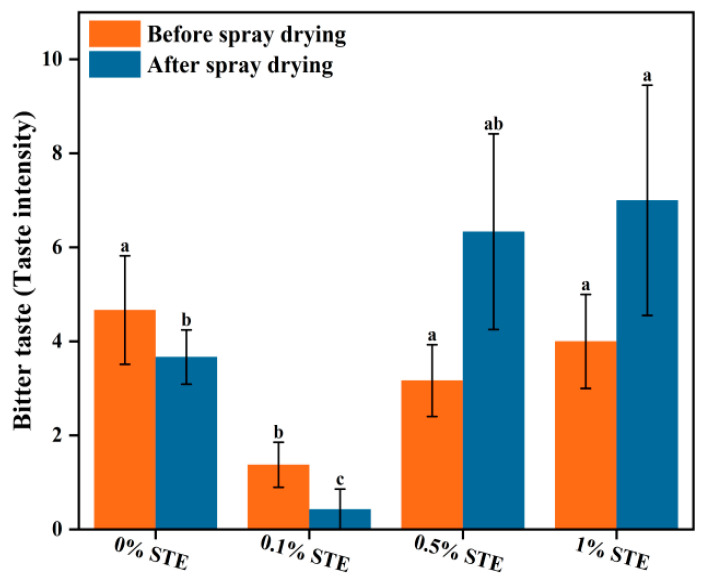
Bitter taste of peptide nanoparticles (ultrasound for 20 min) and SPSS mixed samples before spray drying and re-dissolved peptide microcapsule after spray drying with different STE concentrations. Different letters represent significant differences (*p* < 0.05) between different samples.

To examine the bitter-masking effects of encapsulation by spray drying, the bitter taste of peptide nanoparticles and SSPS mixed samples before spray drying was compared to re-dissolved peptide microcapsule powders after spray drying, as shown in Figure 5. Among all samples, the bitter taste of mixed samples and re-dissolved microcapsules slightly decreased at low STE concentrations (0–0.1%, *w*/*w*), indicating that STE (0–0.1%, *w*/*w*), as a sweetener, exhibits excellent bitter-masking effects. The bitter taste conversely increased at high STE concentrations (≥0.5%, *w*/*w*) due to the bitter aftertaste of STE [32]. The bitterness of STE is mainly attributed to the presence of hydrophobic aglycones in its structure. It is noteworthy that, compared to mixed samples, spray drying with 0.1% STE was remarkably effective in attenuating the bitter taste by fabricating peptide microcapsules. STE plays a synergistic role in decreasing the bitter taste of insoluble peptides as a surface-active natural sweetener. However, the re-dissolved microcapsules exhibited a higher bitter taste than mixed samples at high STE concentrations (≥0.5%, *w*/*w*). Rapid migration of more STE molecules to the surface of atomized droplets, owing to its surface activity, resulted in greater STE distribution on the microcapsule surface, which explains the higher bitter taste. These phenomena were in accordance with the morphological structure and powder properties of peptide microcapsules (Figure 4 and Table 1).

## 4. Conclusions

Insoluble soy peptide aggregates formed during protein hydrolysis were mainly stabilized by hydrophobic interactions. Ultrasound induced the formation of soluble peptide nanoparticles (<200 nm) due to the disruption of predominant hydrophobic interactions and the reassembly of ultrasound-induced unfolded peptide fragments. A gradual reduction in bitter taste was observed for ultrasound-induced peptide nanoparticles with increasing ultrasonic time. Moreover, spray-drying encapsulation using SSPS and STE as wall materials effectively improved the fluidity and wettability of microcapsule powders due to the rapid migration of surface-active STE to the atomized droplet surface, as evidenced by a lower angle of repose and wettability time. Compared to peptide microcapsules without STE, STE-encapsulated peptide microcapsules exhibited lower density and formed spherical particles with smooth surfaces and larger sizes. Moreover, STE (0–0.1%) as a sweetener exhibited excellent bitter-masking effects for peptide microcapsules. However, at high STE concentrations (>0.5%), peptide microcapsules showed a higher bitter taste than unencapsulated peptides due to the greater distribution of STE on the microcapsule surface. Ultrasound-induced reassembly and spray-drying encapsulation of insoluble peptide aggregates may be an efficient strategy for developing functional protein ingredients for application in the food industry.

## Figures and Tables

**Table 1:** Powder properties of peptide microcapsules with different STE concentrations.

Scheme.	Bulk Density (g/mL)	Tap Density (g/mL)	Degree of Compression (%)	Angle of Repose (°)	Wettability (s)
0	0.419 ± 0.01 ^a^	0.718 ± 0.01 ^a^	41.7 ± 1.40 ^a^	48.47 ± 2.04 ^a^	587.00 ± 27.58 ^a^
0.1	0.348 ± 0.01 ^b^	0.621 ± 0.04 ^b^	43.9 ± 3.00 ^a^	46.51 ± 1.63 ^b^	582.67 ± 17.01 ^a^
0.5	0.340 ± 0.02 ^b^	0.594 ± 0.05 ^b^	44.1 ± 2.10 ^a^	40.81 ± 0.71 ^c^	330.00 ± 14.14 ^b^
1.0	0.349 ± 0.01 ^b^	0.621 ± 0.03 ^b^	43.6 ± 3.80 ^a^	40.29 ± 2.18 ^c^	300.67 ± 36.50 ^b^

Different letters represent significant differences between different samples (*p* < 0.05).

## Data Availability

The original contributions presented in the study are included in the article, further inquiries can be directed to the corresponding author.
